# Infusions of scopolamine in dorsal hippocampus reduce anticipatory responding in an appetitive trace conditioning procedure

**DOI:** 10.1002/brb3.1147

**Published:** 2018-10-31

**Authors:** Marie A. Pezze, Hayley J. Marshall, Helen J. Cassaday

**Affiliations:** ^1^ School of Psychology University of Nottingham Nottingham UK

**Keywords:** dorsal hippocampus, rat, scopolamine, trace conditioning

## Abstract

**Introduction:**

Trace conditioning is impaired by lesions to dorsal hippocampus, as well as by treatment with the muscarinic acetylcholine antagonist scopolamine. However, the role of muscarinic receptors within hippocampus has received little attention.

**Methods:**

The present study examined the effects of intra‐hippocampal infusion of scopolamine (30 µg/side) in an appetitive (2 vs. 10 s) trace conditioning procedure using sucrose pellets as the unconditioned stimulus (US). Locomotor activity (LMA) was examined in a different apparatus.

**Results:**

Intra‐hippocampal scopolamine reduced responding to the 2 s trace conditioned stimulus (CS). Intra‐hippocampal scopolamine similarly depressed responding within the inter‐stimulus interval (ISI) at both 2 and 10 s trace intervals, but there was no such effect in the inter‐trial interval. There was also some overall reduction in responding when the US was delivered; significant at the 10 s but not at the 2 s trace interval. A similar pattern of results to that seen in response to the CS during acquisition was shown drug‐free (in the 5 s post‐CS) in the extinction tests of conditioned responding. LMA was increased under scopolamine.

**Conclusions:**

The results suggest that nonspecific changes in activity or motivation to respond for the US cannot explain the reduction in trace conditioning as measured by reduced CS responding and in the ISI. Rather, the findings of the present study point to the importance of associative aspects of the task in determining its sensitivity to the effects of scopolamine, suggesting that muscarinic receptors in the hippocampus are important modulators of short‐term working memory.

## 1 INTRODUCTION

Trace conditioning procedures test the cognitive capacity to associate events separated by an intervening time or “trace” interval. This requires working memory for the moment‐to‐moment processing necessary to bridge the temporal discontiguity (Levy & Goldman‐Rakic, 2000; Sweatt, 2004). Trace conditioning has been shown to be impaired by the use of the muscarinic acetylcholine (ACh) antagonist scopolamine in eyeblink (Kaneko & Thompson, 1997), fear conditioning (Hunt & Richardson, 2007), and appetitive conditioning procedures (Seager, Asaka, & Berry, 1999). The hippocampus has been identified as a key neural substrate of the ability to bridge temporal discontiguity, which is essential to the acquisition of trace conditioning (Bangasser, Waxler, Santollo, & Shors, 2006; Weible, O'Reilly, Weiss, & Disterhoft, 2006). The consolidation or expression of trace fear conditioning (measured after post‐training lesions) has also been found to depend on (dorsal) hippocampus (Quinn, Oommen, Morrison, & Fanselow, 2002). There is moreover some evidence to suggest that muscarinic ACh receptors modulate both the acquisition and expression of trace fear conditioning (Pang et al., 2010). Specifically, injection of the muscarinic antagonist scopolamine in dorsal hippocampus (before the conditioning or the test stages of the procedure) impaired trace fear conditioning (Pang et al., 2010).

The majority of studies conducted to examine the neural substrates of trace conditioning have used aversive eyeblink (Chen et al., 2014; Thomas & Tran, 2012; Weible et al., 2006; Weible, McEchron, & Disterhoft, 2000; Weible, Weiss, & Disterhoft, 2003) or fear conditioning (Bang & Brown, 2009; Bangasser et al., 2006; Baysinger, Kent, & Brown, 2012; Pang et al., 2010; Quinn et al., 2002) procedures. These studies have included examination of the effects of scopolamine administered directly by microinfusion, for example, in perirhinal cortex (Bang & Brown, 2009) and amygdala (Baysinger et al., 2012), in both cases using rats tested with a 16 s trace interval in a conditioned freezing procedure. Similarly, trace fear conditioning was impaired after scopolamine infusion in dorsal hippocampus (Pang et al., 2010), but—to our knowledge—there has been no further examination of the role of scopolamine administered directly into the hippocampus.

Fear conditioning procedures necessarily involve the use of foot shocks. These need not be very aversive in order to motivate learning, but the adoption of appetitive task variants offers the opportunity to further refine procedures. Notably, however, the demonstration of hippocampal lesion effects in appetitive trace conditioning procedures has been challenging (Thibaudeau, Doré, & Goulet, 2009; Thibaudeau, Potvin, Allen, Doré, & Goulet, 2007) and it is important to consider temporal factors such as the timing of conditioned responses (Tam & Bonardi, 2012) as well as the length of the trace interval in relation to the inter‐trial interval (ITI; Chan, Shipman, & Kister, 2014).

Seager et al. (1999) reported that a disruption of appetitive trace conditioning was produced by central cholinergic blockade (following subcutaneous administration of scopolamine hydrobromide but not scopolamine methyl bromide) and electrophysiological responses provided evidence that this effect was mediated in hippocampus. However, these findings have yet to be confirmed by direct infusion of scopolamine into hippocampus. To address these gaps in the evidence base, the present study therefore set out to test the importance of cholinergic signaling in hippocampus using an appetitive trace conditioning procedure.

Specifically, we examined the effects of scopolamine infused into dorsal hippocampus (30 µg/side; Pang et al., 2010) in an appetitive procedure identical to that which we have used previously to compare conditioning at 2 s and 10 s trace intervals following microinfusions in prefrontal cortex (mPFC) sub‐regions (Pezze, Marshall, & Cassaday, 2015, 2017). We also tested the effects of the same scopolamine treatment on locomotor activity (LMA).

As noted above, the replication of the effects of hippocampal lesions, reliably shown in aversive trace conditioning, has been challenging when appetitive procedures are used (Chan et al., 2014; Tam & Bonardi, 2012; Thibaudeau et al., 2007, 2009). We therefore sought to test the generality of the finding that intra‐hippocampal scopolamine impairs trace conditioning (Pang et al., 2010), using the same appetitive procedure which previously showed sensitivity to scopolamine administered systemically (as well as in mPFC; Pezze, et al., 2017).

Based on these previous findings from our laboratory, it was anticipated that scopolamine would impair trace conditioning at the 2 s trace interval. Previous findings suggest that floor effects can preclude the demonstration of any further impairment in trace conditioning at the 10 s trace interval. The use of the 10 s trace was nevertheless retained in the present study because conditioning at the higher level of temporal discontiguity would be expected to be more dependent on hippocampal function. Other appetitive trace studies (e.g., Chan et al., 2014; Thibaudeau et al., 2007) have compared short and long trace intervals similar to those used in the present experiment. Moreover, we also routinely examine the distribution of responding within the longer 10 s trace interval (Pezze et al., 2015, 2017; Tam & Bonardi, 2012). Extinction tests were conducted drug‐free as a further control measure, for scopolamine effects on motor responding. Delay conditioning groups were not included because appetitive delay conditioning is not impaired by hippocampal lesions (Thibaudeau et al., 2009, 2007), and the available evidence shows no effect of scopolamine on (in this case, aversive) delay conditioning (Pang et al., 2010; Pezze et al., 2017).

## 2 MATERIALS AND METHODS

### 2.1 Subjects

Forty experimentally naïve male Wistar rats were used (Charles Rivers, UK; 170–213 g; mean 191 g). Two of the subjects died under anesthetic due to surgical complications, and a third was humanely euthanized after the third day of conditioning because of nonspecific symptoms that might have developed in severity. Rats were weighed daily following surgery, and all the other animals used in the present study recovered well.

Throughout the study, rats were housed in groups of 3–4 per cage, on a 12:12‐hr dark/light cycle and on ad libitum water. Food was freely available up until 24 hr before shaping. Rats were handled for approximately 10 min/day over the course of one week, during which time they reached full adult weight (261–299 g; mean 280 g) prior to surgery, which was also conducted over 1 week. Shaping commenced after at least 1 week of post‐operative recovery, throughout which rats were monitored daily and habituated to the manual handling techniques that would be used to restrain them during the microinfusion procedure (weights at the start of food deprivation 297–376 g; mean 335 g). Weight gain was variable post‐operatively, but because we used vehicle‐injected controls, all the rats had the same experience aside from the infusion of scopolamine over the 4 days of conditioning. Allocations to experimental (drug and trace) groups were weight‐matched as far as possible.

In total, 37 rats completed the trace conditioning procedures and 24 of the same rats were subsequently tested in the LMA chambers during the week following the appetitive conditioning (i.e., 4 days after the completion of the extinction tests). Animals were selected to be (as far as possible) weight‐matched in the vehicle‐ and scopolamine‐infused groups as well as counterbalanced for their previous infusion condition.

All procedures were carried out in accordance with the principles of laboratory animal care, specifically the United Kingdom (UK) Animals Scientific Procedures Act 1986, Project Licence Number: PPL 40/3716. Under this licence, we are required to maintain adult rats in the range 200–300 g, this range increasing to 300–400 g for older cohorts, and any drop below 80% initial free‐feeding weight requires compensatory feeding with wet mash. Our criteria for recovery from the surgery before any food restriction is introduced include a 10% increase in body weight; the weight gain was a minimum of 13% for this particular batch of animals.

### 2.2 Apparatus

Four fully automated conditioning boxes housed within sound‐attenuating cases containing ventilation fans (Cambridge Cognition, Cambridge, UK) were used. The conditioning boxes were steel (25 cm × 25 cm × 22 cm high) with a Plexiglas door (27 cm × 21 cm high), inset at the front. The food magazine (recessed in a side wall of each of the chambers) was constantly illuminated whenever food was available and accessed via a flap. Nose‐pokes were recorded by the breaking of the photobeam within the food magazine. The unconditioned stimulus (US) was two 45 mg sucrose pellets dispensed into the magazine (Formula F, Noyes Precision Food, New Hampshire, UK). The conditioned stimulus (CS) was a mixed frequency noise, presented via a loudspeaker in the roof of the chamber, set at 72 dB including background and of 5 s duration.

Additionally, an experimental background stimulus was provided by three wall‐mounted stimulus lights and the house light flashing on (0.5 s) and off (0.5 s), continuously for the duration of the conditioning session. Stimulus control and data collection were by an Acorn Archimedes RISC computer programmed in Basic with additional interfacing using an Arachnid extension (Cambridge Cognition).

Locomotor activity was measured in a dimly lit (50–70 Lux) room in 12 clear Perspex chambers (39.5 cm long × 23.5 cm wide × 24.5 cm deep) with metal grid lids. The chambers were surrounded by frames containing two levels of photobeams (Photobeam Activity System, San Diego Instruments, USA) as described previously (Jones, Brown, Auer, & Fone, 2011; Pezze, Marshall, Fone, & Cassaday, 2015; Pezze et al., 2017).

### 2.3 Implantation of guide cannulae into the dorsal hippocampus

Rats were anesthetized using isoflurane delivered in oxygen (induction: 4%–5%; maintenance: 1%–3%) and secured in a stereotaxic frame. All surgical procedures were carried out under aseptic conditions: A sterilized scalpel blade was used to expose the skull, and burr holes were drilled to allow the implantation of bilateral infusion guide cannulae (3.0 mm apart).

To reduce the risk of animals experiencing pain upon recovery from the general anesthetic, the incision was treated with the local anesthetic lidocaine and all rats were injected with the nonsteroidal anti‐inflammatory Rimadyl (1:9; 0.8 ml/kg, s.c.). The tips of the guide cannulae were aimed 0.5 mm above the injection sites in dorsal hippocampus. The specific coordinates were ±1.5 mm lateral, −3 mm posterior from bregma, and −3.5 mm ventral from the skull surface. The cannulae were secured using four stainless steel screws and dental acrylic. To reduce the risk of infection, plastic stylets were inserted into the tubes and closed with a plastic dust cap. Rats were placed in a recovery incubator before being reintroduced to their cages. Because of the previous incidence of meningitis (Pezze et al., 2015, 2017), the antibiotic Synulox (Ready‐To‐Use, Pfizer Ltd; 0.05 ml kg^‐1^ day^‐1^, s.c.) was administered as a prophylactic measure.

### 2.4 Microinfusion procedure

Scopolamine was prepared in a concentration of 60 µg/µl saline for injection in 0.5 μl volumes to deliver a dose of 30 µg/side. Rats were carefully restrained whilst the injectors were inserted into the cannulae guides into the dorsal hippocampus for direct infusion. Two lengths of polythene tubing were used to connect the injection syringes to the injectors. The two syringes were mounted on a motorized microinfusion pump, programmed to deliver 0.5 μl of scopolamine or saline vehicle, bilaterally over 2 min, plus an additional 1 min after infusion for further diffusion. The four conditioning sessions started 10 min after the infusions during which time the rats were monitored for adverse effects following the infusion (none was detected). LMA tests followed immediately on completion of the infusion, to document the onset and course of scopolamine effects.

### 2.5 Trace procedure

One day prior to shaping, food was removed in the morning and the rats were introduced to sucrose pellets in the home cage later the same day before being fed standard laboratory chow rationed at 5 g/100 g body weight. Behavioral procedures were identical to those used to examine the effects of scopolamine microinfused in mPFC (Pezze et al., 2017). Each rat was repeatedly infused with either saline or scopolamine prior to each of the four conditioning sessions; the rats were not infused prior to the two extinctions session. The trace procedure is summarized in Table 1.

**Table 1:** The trace conditioning schedule by experimental day, drug treatment, and stimuli presented within the conditioning boxes. Following two days shaping to accustom rats to eating from the magazine, pre‐1 and pre‐2 refer to the baseline sessions conducted with automated unsignaled food deliveries. D1 to D4 refer to the 4 days of trace conditioning, on each of which tone was followed by food at the 2 s or 10 s trace interval, for a 30 trials per day and in the presence of the experimental background (flashing lights) stimulus. Tests 1 and 2 refer to the presentation of the noise and flashing lights (in counterbalanced order) to provide extinction measures of the strength of the associations

Conditioning day	Drug treatment	Stimuli
Shaping	Drug‐free	Food
Shaping	Drug‐free	Food
Pre‐1 baseline	Drug‐free	Food
Pre‐2 baseline	Drug‐free	Food
Trace—D1	Scopolamine	30 × tone‐food (within flashing light)
Trace—D2	Scopolamine	30 × tone‐food (within flashing light)
Trace—D3	Scopolamine	30 × tone‐food (within flashing light)
Trace—D4	Scopolamine	30 × tone‐food (within flashing light)
Test 1	Drug‐free	Tone or flashing light
Test 2	Drug‐free	Flashing light or tone

### 2.6 Preconditioning

There were two days of shaping to accustom rats to eating from the magazine (starting with the tray flaps propped open by a teaspoonful of sucrose pellets; and conducted in pairs of cage mates and with the outer case door open until each rat was noted to take food). There then followed two days of baseline sessions, during which there were 30 unsignaled rewards (each of two sucrose pellets) over 60 min, delivered on a variable interval around 120 s, to habituate rats to the sounds produced by food delivery.

### 2.7 Conditioning

There were 4 days of conditioning at 30 trials per day. Depending on the experimental group, the sucrose pellet reward (US) was delivered at a 2 s or 10 s inter‐stimulus interval (ISI), measured from CS offset to US delivery (in the two different trace groups). On each conditioning day, 30 signaled rewards were presented on a variable interval around 120 s, with the constraint that the ITI was always at least 1.5 times longer than the ISI length, that is, minimum 3 s for the 2 s and 15 s for the 10 s trace conditioned groups. Although the minimum ITI depended on trace condition in use, the average ITI was the same for the 2 s and 10 s trace groups because in each case the trace interval in use added to the overall duration of a 60‐min session (taking this to 61 or 65 min in total, respectively). Throughout acquisition, the background stimulus (flashing lights) was presented continuously. As per previous studies, this continuous presentation also encompassed the 2 s or 10 s ISI, as applicable (Pezze et al., 2015, 2017).

Extinction tests of conditioning to the CS (noise) and experimental background (flashing lights) stimuli were conducted in a counterbalanced order, 24 and 48 hr after conditioning procedures had been completed. In each case, there were thirty 5 s presentations of the stimulus under test, over the course of a 60‐min session. Stimuli were presented at a 120 s variable interval (the same for all groups, without the minimum related to the trace interval in use as per the conditioning stage).

### 2.8 Locomotor activity

Two consecutive breaks of adjacent beams within the lower level of photobeams generated a locomotor count. To start a session, rats were placed into the center of the chamber. On the day before the LMA tests, each rat was habituated to its allocated activity chamber for 30 min (drug free). Then, on the test day there was a further 30‐min habituation. Each rat was then removed for drug treatment and then immediately replaced in its allocated LMA chamber. Total locomotor counts were recorded for each consecutive 10‐min epoch for a further 60 min.

### 2.9 Design and analysis

Four between‐subjects experimental groups were specified by the trace condition (2 or 10 s) and drug (saline or scopolamine microinfusion). The analyses of variance (ANOVAs) were run as mixed factorial designs using SPSS, with within‐subjects factors related to the passage of time (days, blocks of trials within day 1, bins within the trace). Specifically, there was a repeated‐measures factor of days (at four levels) for acquisition, which was examined in 2 × 2 × 4 designs. Effects on the initial acquisition of responding to the CS were further examined on day 1, in relation to blocks of five trials (at six levels) in a 2 × 2 × 6 design.

The dependent variable was the number of nose‐pokes into the food magazine (recorded as totals) in different periods of the conditioning sessions. Thus, the same dependent variable was used to index anticipatory responding during the 5 s presentations of the CS and in the ISI trace interval. To try to distinguish effects on motivation for food reward or motor responding, similar analyses were conducted on consummatory responding during the 5 s following US delivery as well as on ITI responding in the remainder of the session (excluding the trace ISI). Responding in the variable ITIs was summed over the session and captured as total “residual” responding. We also looked at ITI responding in the 5 s immediately pre‐CS, for a comparison measure of responding just before each trial. However, the response rates in the 5 s ITI immediately pre‐CS were very low and unsuitable for ANOVA.

Additionally, the responding of the animals during the trace interval between CS offset and US delivery was also examined, separately for the 2 s and 10 s conditioned groups as the intervals in use were not directly comparable. Moreover, the 10 s ISI was broken down into five 2 s bins of time and analyzed using repeated‐measures ANOVAs by bins as well as days, with the between‐subjects factor of drug. These analyses were 2 × 5 × 4 because trace was no longer a factor.

Extinction day test results were examined using 2 × 2 multivariate ANOVAs, to examine the effects of prior infusion of scopolamine by trace condition on total responding, during the CS, 5 s post‐CS, and in the remainder of the ITI. The equivalent analyses were also run for the extinction session testing responding to the background light stimulus. As per the early acquisition data, the course of extinction was similarly examined over six blocks of 5 s presentations, for both the previously trained CS and the background light stimulus, in 2 × 2 × 6 ANOVAs.

For interactions between scopolamine infusion (drug) and the repeated‐measures factors, we report the results of the within‐subjects contrasts (trend analyses). Differences relating to drug group were also followed up by post hoc tests using Fisher's LSD test.

The LMA data were analyzed by mixed ANOVAs with the between‐subjects factor of drug (saline or scopolamine microinfusion) by blocks of 10 min, at three levels for the 30‐min test day habituation, and six levels for the 60‐min testing under drug, in 2 × 3 and 2 × 6 designs, respectively. The dependent variable was beam breaks.

## 3 RESULTS

**Figure 1:**
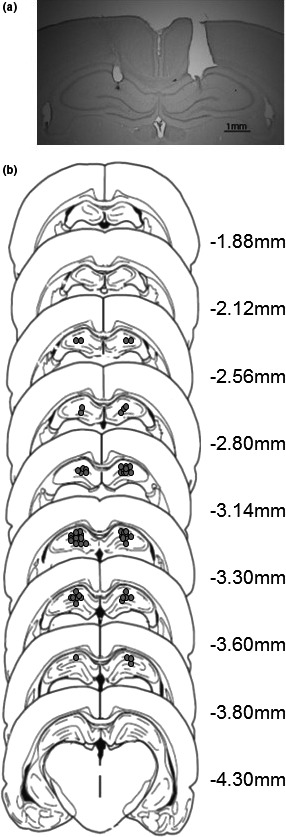
(a) Photograph of a representative placement which illustrates the area around the injection which is also representative of the degree of gliosis seen as a result of the microinfusions. (b) Approximate locations of infusion cannula tips, in the dorsal hippocampus. Placements are shown on coronal plates adapted from Paxinos and Watson (1998), with numbers indicating distance from bregma in millimeters

No rats needed to be excluded on histological grounds, leaving a final sample size of 37 (*n* = 9–10/group). The number of infusions was 4–5, because 24 of the same rats (which had been infused for the 4 days of conditioning) were subsequently tested in LMA, requiring an additional infusion. Figure 1a shows a representative histological section; there was little evidence of damage at the cannula tip. Figure 1b illustrates the full range of cannula placements.

There was some missing data capture (four rats’ data from the second preconditioning day; and the breakdown by trial blocks was lost for three rats’ CS extinction tests).

### 3.1 Effects of scopolamine in dorsal hippocampus on appetitive trace conditioning

#### 3.1.1 Preconditioning (drug free)

All the reward pellets were consumed, and the rats were confirmed to be nose‐poking freely following the shaping procedure. There were no effects involving drug condition‐to‐be on responding in the individual baseline sessions, maximum *F*(1,30) = 2.785, *p* = 0.106.

#### 3.1.2 ITI responding (following infusions)

**Figure 2:**
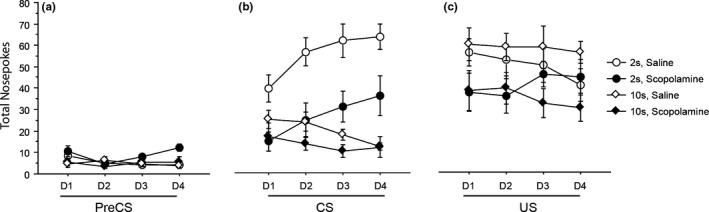
Mean nose‐pokes are shown as a function of the 4 days of conditioning (D1 to D4) for (a) the 5 s of the inter‐trial interval (ITI) just prior to conditioned stimulus (CS) presentation (pre‐CS) as compared to those (b) during the 5 s CS presentation or (c) in the 5 s when food was delivered (US). Diamonds denote rats conditioned at the 10 s trace interval, and circles denote rats conditioned at the 2 s trace interval after bilateral scopolamine infusion (30 µg in 0.5 µl/side) in dorsal hippocampus (black fill). White fill denotes control groups of rats conditioned after microinfusion of saline vehicle. *N* = 9–10 rats per group. Error bars represent the standard error of the mean

There were no effects involving drug, and so no evidence for any reduction of responding after microinfusion of scopolamine, in the ITI, maximum *F*(3,99) = 2.020, *p* = 0.116. In the ITI, there was just a main effect of days, *F*(3,99) = 5.791, *p* = 0.001, because of an overall reduction in responding over the 4 days of testing (day 1 *M* = 177.258 ± 16.725; day 4 *M* = 135.497 ± 13.953). Figure 2 (panel a) shows the 5 s ITI just preceding the 5 s CS presentations, for direct comparison with subsequent CS and US responding. As can be seen in the figure, the response rates in the 5 s ITI immediately pre‐CS were very low and unsuitable for ANOVA.

#### 3.1.3 CS responding (following infusions)

Rats conditioned at the 2 s trace responded progressively more to the CS over the 4 days of testing (day 1 *M* = 27.033 ± 3.930; day 4 *M* = 49.800 ± 4.271) whilst rats conditioned at the 10 s trace responded progressively less (day 1 *M* = 20.944 ± 4.032; day 4 *M* = 12.167 ± 4.382). There was a significant trace × days interaction, *F*(3,99) = 11.194, *p* < 0.001, as well as a main effect of trace *F*(1,33) = 27.792, *p* < 0.001. There were no drug effects involving days, but there was both a main effect of drug, *F*(1,33) = 14.562, *p* = 0.001, and a drug × trace interaction, *F*(1,33) = 5.684, *p* = 0.023. As shown in Figure 2 (panel b), responding to the CS was overall reduced under scopolamine and this effect was significant for the 2 s, *p* < 0.001, but not the 10 s trace conditioned groups, *p* = 0.325. Within day 1, the analysis by trial blocks showed an interaction between blocks and drug, *F*(5,170)=4.408, *p* = 0.001, consistent with the possibility that scopolamine microinfusions depressed the course of early acquisition, and this effect was also significant in the linear trend, *F*(1,43) = 11.435, *p* = 0.002. However, there was no effect of drug × trace × blocks, *F*(5,170) = 1.139, *p* = 0.342, so the daily averages are shown in Figure 2b.

#### 3.1.4 US responding (following infusions)

Responding overall dropped from day 1 (*M* = 47.700 ± 4.022) to day 4 (*M* = 42.706 ± 3.485) except in the scopolamine 2 s group, which showed some increase from a lower baseline (day 1 *M* = 37.333 ± 8.147; day 4 *M* = 44.333 ± 7.059). Statistically, there was a trace × drug × days interaction, *F*(3,99) = 3.084, *p* = 0.031, significant also in the linear trend, *F*(1,33) = 6.664, *p* = 0.014. As shown in Figure 2 (panel c), responding following US deliveries was overall reduced under scopolamine. Statistically, this was reflected in a main effect of drug, *F*(1,33) = 5.613, *p* = 0.024. However, in contrast to the effect on CS responding, this effect was significant for the 10 s, *p* = 0.023, but not the 2 s trace conditioned groups, *p* = 0.354. Importantly, and irrespective of trace condition, pellets were nonetheless found to be eaten at the end of each conditioning session.

#### 3.1.5 ISI responding (following infusions)

##### 2 s trace group

**Figure 3:**
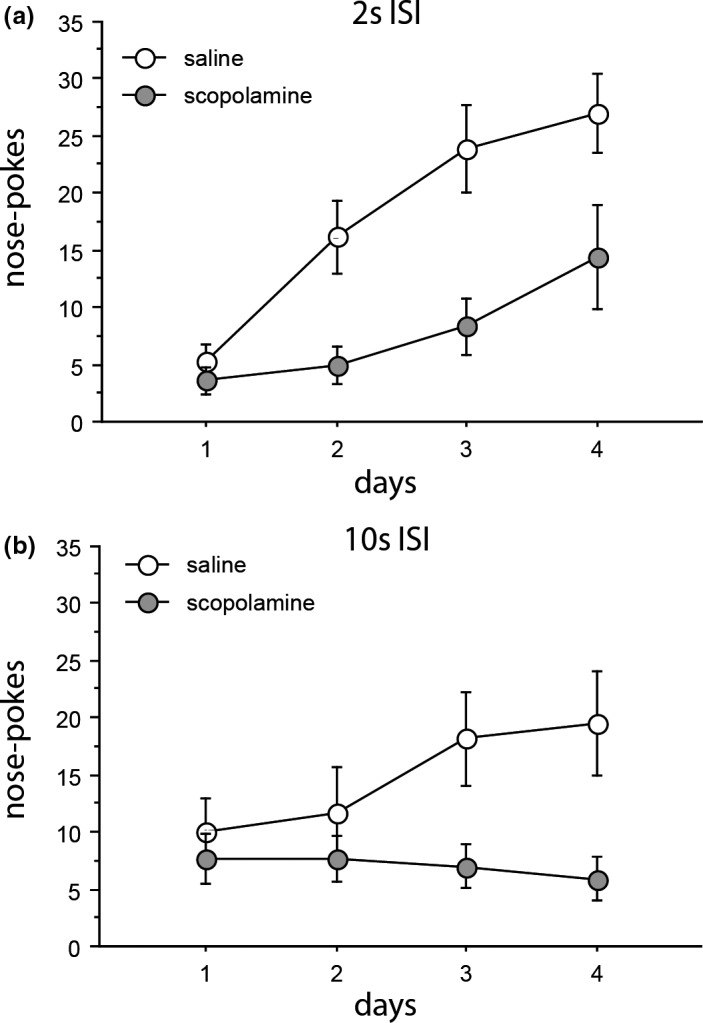
Mean total nose‐pokes are shown as a function of the 4 days of conditioning (a) during the 2 s inter‐stimulus interval and (b) during the 10 s inter‐stimulus interval. Gray circles denote rats conditioned after bilateral microinfusion into dorsal hippocampus of scopolamine at (30 µg in 0.5 µl/side) or saline vehicle. White circles denote rats conditioned after microinfusion of saline vehicle. *N* = 9–10 rats per group. Error bars represent the standard error of the mean

There was a main effect of days, *F*(3,51) = 28.407, *p* < 0.001, and a drug x days interaction, *F*(3,51) = 6.875, *p* = 0.001, which was also significant in the linear trend, *F*(1,17) = 7.637, *p* = 0.013. There was also a main effect of drug, *F*(1,17) = 22.375, *p* < 0.001. Responding in the 2 s ISI progressively increased over the 4 days of conditioning, but this effect was less marked in rats treated with scopolamine (Figure 3a).

##### 10 s trace group

There was an effect of bins because responding was not uniformly distributed within the 10 s trace, *F*(4,192) = 2.779, *p* = 0.034, with some relatively increased responding toward the middle to later portion of the trace interval but not the final bin, suggesting that timing was not fully developed (bin 1 *M* = 1.956 ± 0.422; bin 2 *M* = 2.333 ± 0.261; bin 3 *M* = 2.113 ± 0.385; bin 4 *M* = 2.791 ± 0.450; bin 5 *M* = 1.833 ± 0.362). However, (the acquisition of) this distribution was unaffected by drug, which was significant only as a main effect, *F*(1,16) = 5.467, *p* = 0.033. Scopolamine‐treated rats responded overall less in the 10 s ISI (Figure 3b). In contrast to what was seen in the 2 s trace group, the drug x days interaction was not significant, *F*(3,48) = 2.019, *p* = 0.124, in the linear trend, *F*(1,16) = 4.344, *p* = 0.054. There was no interaction between drug and bin, *F*(4,64) = 1.158, *p* = 0.338.

#### 3.1.6 Extinction tests (drug free)

##### Noise CS

There was no overall effect of prior trace or drug group on overall responding to the noise CS during the extinction tests, maximum *F*(1,32) = 2.814, *p* = 0.103. The analysis by extinction trials within the one‐day session showed the expected effect of blocks, *F*(5,150) = 13.405, *p* < 0.001, because responding reduced as the session progressed (block 1 *M* = 3.937 ± 0.494; block 5 *M* = 1.285 ± 0.263). However, there were no significant interactions involving prior drug group and block, both *F*s <1. Neither were there any effects involving drug in the ITI, maximum *F*(1,32) = 1.736, *p* = 0.197. There were, however, main effects of both conditioning trace, *F*(1,32) = 20.663, *p* < 0.001, and prior drug on responding in the 5 s after noise presentations (post‐CS), *F*(1,32) = 7.180, *p* = 0.012, as well as a drug × trace interaction, *F*(1,32) = 6.139, *p* = 0.019. The pattern of effects was similar to that seen on CS responding during acquisition: Responding post‐CS was reduced in rats previously conditioned under scopolamine. As shown in Table 2A, this difference between scopolamine‐ and saline‐infused groups was clear in the 2 s trace conditioned group, *p* = 0.001. The post‐CS difference between the saline versus scopolamine 10 s trace conditioned groups was not significant, *p* = 0.890.

**Table 2:** Mean total nose‐poke responding (±*SEM*) during different parts of the extinction sessions for (A) the noise conditioned stimulus (CS) and (B) the light background stimulus, by the previously conditioned trace and drug infusion groups. The CS or light background presentations were each of 5 s duration. Responding post‐CS or post‐stimulus was similarly recorded over a 5 s duration. Responding in the remainder of the session was in the inter‐trial interval (ITI). *N* = 8–10/cell

Trace	2 s		10 s	
Drug infusion	Saline	Scopolamine (30 µg/side)	Saline	Scopolamine (30 µg/side)
(A)				
Tone CS	14.300 (±2.175)	12.778 (±2.293)	7.250 (±2.432)	12.111 (±2.293)
Post‐CS	16.200 (±1.626)	7.333^***^ (±1.714)	4.125 (±1.818)	3.778 (±1.714)
ITI	83.100 (±15.457)	120.222 (±16.293)	98.375 (±17.281)	105.556 (±16.293)
(B)				
Light background	3.400 (±0.991)	1.667 (±1.045)	3.625 (±1.108)	2.000 (±1.045)
Post‐stimulus	3.300 (±0.799)	2.667^#^ (±0.842)	4.375 (±0.893)	1.444# (±0.842)
ITI	82.300 (±20.704)	96.778 (±21.824)	93.375 (±23.148)	66.111 (±21.824)

Note

Asterisks indicate a significant difference by prior infusion group as compared to the corresponding 2 s‐conditioned saline group with ^***^ *p* = 0.0001. Hash signs indicate the overall effect of having been conditioned under scopolamine with ^#^ *p* < 0.05.

##### Light background

As per the noise CS, there was no overall effect of prior trace or drug group on total responding to the light stimulus during the extinction tests, maximum *F*(1,32) = 2.568, *p* = 0.119. The analysis by trials showed some effect of blocks, *F*(5,165) = 2.124, *p* = 0.065, significant in the linear trend, *F*(1,33) = 5.286, *p* = 0.028. As might be expected, responding to the light stimulus was less than that seen to the noise CS; nonetheless, from a lower baseline, responding reduced as the session progressed (block 1 *M* = 0.758 ± 0.229; block 5 *M* = 0.161 ± 0.090). As was the case for the CS responding, there were no significant interactions involving prior drug group and block, both *F*s <1. However, ANOVA of responding during the light tests did show a main effect of prior drug post‐stimulus, *F*(1,33) = 4.915, *p* = 0.034, in the absence of any other significant effects or interactions, maximum *F*(1,33) = 2.235, *p* = 0.144. Responding post‐stimulus was overall reduced in rats previously conditioned under scopolamine (Table 2B).

#### 3.1.7 Effects of scopolamine in dorsal hippocampus on locomotor activity

**Figure 4:**
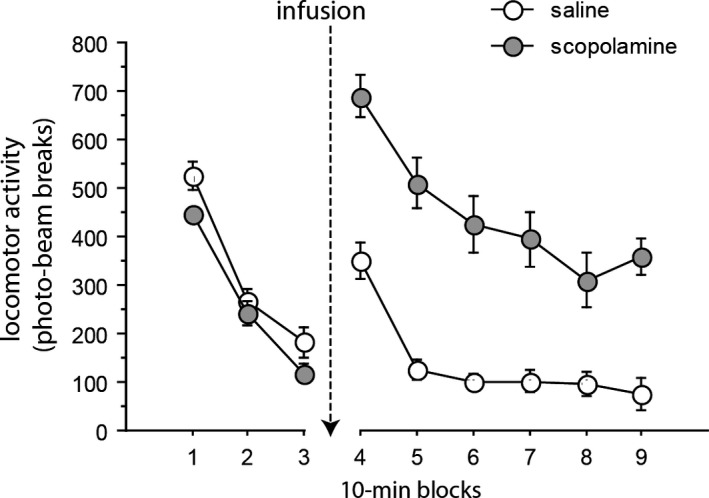
Effect of microinfusion of scopolamine on spontaneous activity. Rats were habituated to the activity chambers for 30 min before bilateral microinfusion of scopolamine at 30 µg in 0.5 µl/side (gray circles) or saline vehicle (white circles). Locomotor activity was then monitored for an additional 60 min. *N* = 12 rats per group. Error bars represent the standard error of the mean

Groups were not evenly matched in that there was an effect of drug condition‐to‐be during the test day habituation, *F*(1,22) = 4.926, *p* = 0.037. This effect arose because rats allocated to the saline infusion condition were initially more active (*M* = 324.667 ± 17.859) than those allocated to the scopolamine condition (*M* = 268.611 ± 17.859). However, post‐infusion, the effect of drug *F*(1,22) = 44.802, *p* < 0.001, took the form that scopolamine‐treated rats were more active (*M* = 447.903 ± 32.373) than those infused with saline (*M* = 141.458 ± 32.373). As shown in Figure 4, this effect was consistent across the test session in that whilst there was a main effect of blocks, *F*(5,110) = 35.973, *p* < 0.001, the drug‐by‐block interaction was not significant, *F*(5,110) = 2.237, *p* = 0.056.

## 4 DISCUSSION

Intra‐hippocampal infusion of scopolamine (within the range of infusion sites shown in Figure 1) reduced responding to a trace conditioned CS, an effect most clearly seen when the food US (two sucrose pellets) was presented at a 2 but not at a 10 s trace interval (possibly because of a floor effect in conditioning to the CS presented at 10 s; Figure 2b). The same pattern of results was confirmed drug‐free in the extinction tests of conditioned responding rendering direct nonspecific effects of the scopolamine treatment an unlikely account of trace conditioning impairment (post‐CS, Table 2A). Moreover, in contrast to its effects in the ISI (Figure 3), responding in the ITI was unaffected by scopolamine infusion. In a separate measure in a different apparatus, LMA was increased under scopolamine (Figure 4). Taken together, these data suggest that nonspecific changes in motor activity provide no account of reduced trace conditioning as measured by reduced CS responding.

There was also some overall reduction in responding immediately after the sucrose pellet US was delivered. However, as Figure 2c shows, responding was nonetheless sufficiently high to ensure collection of the 30 US deliveries over the course of the conditioning session (and none was left at the end of the session). Additionally, the effect of scopolamine on US responding was different to that seen in the case of the CS in that the reduction in US responding was significant at the 10 s but not at the 2 s trace interval. Thus, motivational differences, specifically loss of interest in the sucrose US after scopolamine infusion, do not provide a compelling account of the differences in CS responding.

The drug‐free extinction sessions provide further evidence as to whether scopolamine interfered with the acquisition and/or consolidation of the CS‐US association as distinct from the ability to perform the nose‐poke response during acquisition. In the previous fear conditioning study (Pang et al., 2010), intra‐hippocampal scopolamine reduced freezing CRs, even in a hippocampus‐independent delay conditioning group. The authors discussed the possibility that this was due to increased locomotion, shown also in the present study, which could interfere with freezing or (in the present study) nose‐poking for food. The CS data from the extinction session did not show any effect of prior trace or drug group (Table 2A). The overall reduced responding during the extinction tests can be attributed to the change in context (due to the absence of the after‐effects of the infusions and the fact that food was no longer available). Nonetheless, analysis of post‐CS responding suggested impaired expression of appetitive trace conditioning in rats previously conditioned under scopolamine at the 2 s ISI.

The introduction of a trace interval allows for more than the passage of time and the need for a memory trace in that contextual cues intervene between CS offset and US delivery and may help to bridge the gap in normal animals (Quinn et al., 2002). Moreover, as per our earlier studies, we used an experimental background stimulus to provide an additional measure of effects on contextual conditioning as assessed in drug‐free extinction tests (Pezze et al., 2015, 2017). In the present study, intra‐hippocampal scopolamine also reduced responding after test presentations of the light experimental background (Table 2B). This action is consistent with impaired contextual conditioning to this aspect of the context. However, we found a different profile of effects in the ISI and the ITI during acquisition. There was no reduction in ITI responding after intra‐hippocampal scopolamine. In the ISI, saline‐infused rats showed increased responding over the course of conditioning, and the acquisition of anticipatory responding within the trace intervals was depressed under scopolamine (Figure 3). Different profiles of responding in the ITI and ISI suggest that rats treated with scopolamine discriminated the ISI from the ITI. The effect of intra‐hippocampal scopolamine in the ISI resembled the effect on associative learning: depressed anticipatory responding during CS presentations.

Thus, the results suggest that nonspecific changes in activity or motivation to respond for the US cannot explain the reduction in trace conditioning as measured by reduced CS responding and in the ISI. Rather, the findings of the present study point to the importance of associative aspects of the task in determining its sensitivity to the effects of scopolamine, because these were different by trace and depended on the period of the conditioning session under analysis (i.e., whether CS, US, ISI, or ITI). These findings further suggest that muscarinic receptors in the hippocampus are important modulators of short‐term working memory of the kind required to hold information “online” (Levy & Goldman‐Rakic, 2000; Sweatt, 2004). Before considering the wider implications of the findings, we first discuss the limitations of the present study.

### 4.1 Were the conditioning parameters appropriate to test the role of hippocampus?

It has long been suggested that temporal discontiguity is the critical factor, which renders trace conditioning reliant on the hippocampus (Rawlins, 1985; Wallenstein, Eichenbaum, & Hasselmo, 1998) and there is evidence in support of this contention (Bangasser et al., 2006). In the present study, the use of the 10 s trace was intended to increase the level of temporal discontiguity, a manipulation which might be expected to make trace conditioning more dependent on hippocampal function. However, in the present study the effect of scopolamine infusion in the dorsal hippocampus was demonstrated at the short 2 s rather than at the long 10 s trace interval.

Chan et al. (2014) showed the importance of the trace interval in relation to the ITI for the demonstration of trace conditioning in normal animals. Therefore, these temporal factors can also be important determinants of whether effects of hippocampal lesions may be demonstrated. Thibaudeau et al. (2007) earlier examined the performance of normal rats when the trace was systematically extended up to 8 s, finding that normal rats did not learn at an 8 s trace interval even after 200 trials. However, Chan et al showed than intact rats can learn trace conditioning with ISIs of 8 s and even 60 s, with successful conditioning at 8 s requiring an ITI of 300 s.

Thus, the work of Thibaudeau et al. (2007) and Chan et al. (2014) suggests that our ITI was likely too short to support conditioning at the longer 10 s trace interval. However, we had the constraint that we were working with an earlier established method adapted for microinfusion studies to deliver 30 trials within just over 1 hr (over which time the majority of drug treatments can be assumed to maintain their effectiveness). Moreover, we have previously found that (at least in the 2 s trace conditioned group) learning is reliably shown within 4 days. It was advisable to keep our conditioning parameters consistent to allow direct comparison between experiments, in particular an earlier study showing that appetitive but not aversive trace conditioning was impaired by systemic scopolamine (Pezze et al., 2017).

A further limitation arising from the adoption of the previously used design and procedural parameters is that we did not include delay conditioning or explicitly unpaired groups. We previously employed delay conditioning controls in the aversive but not in the appetitive procedure used to examine the effects of systemic scopolamine (Pezze et al., 2017). However, the appetitive procedure has a number of inbuilt controls for nonspecific effects. For example, differences in unconditioned effects would be expected to be equivalent across behavioral conditions. We are interested in differences by trace interval. Moreover, other appetitive trace studies of the hippocampus (e.g., Chan et al., 2014; Thibaudeau et al., 2007, 2009) have specifically compared short and long trace intervals similar to those used in this experiment.

### 4.2 Were the conditioning parameters appropriate to examine (effects on) timing?

In principle, we might have identified effects on timing since we measured the distribution of the responding within the 10 s trace in the present study. Tam and Bonardi (2012) found that whilst excitotoxic lesions to dorsal hippocampus were without effect on the acquisition of appetitive trace conditioning, they selectively affected the distribution of conditioned responding to the trace CS in the early stages of acquisition and impaired the timing of CRs as measured in the distribution of CRs during the trace CS presentations. Specifically, hippocampal lesioned rats underestimated the time of US delivery and responded earlier than the sham‐lesioned controls. Tam and Bonardi (2012) suggest that a failure to examine changes in the timing of CR production may account for the lack of hippocampal lesion effects in earlier appetitive studies, since the majority of these studies have failed to examine *when* the CR will occur as is more typical in eyeblink conditioning studies. The focus on averaged responding may show a negative result because the overall level of responding to the trace CS is unaffected. Moreover, the effect of hippocampal lesions on CR timing was transient, restricted to the first half of acquisition (Tam & Bonardi, 2012).

In the present study, we did not find any evidence to suggest impaired timing within the trace interval: Statistically, there was no interaction between drug infusion and the 2 s bins of the 10 s trace interval. However, it should be acknowledged that the conditioning schedule used in the present study may have been insufficient for the vehicle controls to show the development of timing in the 10 s trace ISI. We earlier examined the effects of systemic drug treatments on (the development of) the distribution of responding within the ISI of this kind of procedure. After 5 days’ acquisition at 10 trials/day, there were no significant effects of bins (Cassaday, Finger, & Horsley, 2008). However, effects of bins (moderated by systemic drug treatments) were seen when conditioning was conducted over 10 days’ acquisition at 10 trials/day (Cassaday et al., 2008; Kantini, Norman, & Cassaday, 2004). Subsequent microinfusion studies have used a more intensive conditioning schedule identical to that adopted in the present study (conducted over 4 days, each of 30 trials, in order to limit the number of microinfusion sessions; Pezze et al., 2015, 2017). Responding increased within the 10 s ISI over the course of acquisition and scopolamine depressed ISI responding (both systemic and following microinfusion in prefrontal cortex; Pezze et al., 2017). However, there were no significant interactions between treatment and bins for responding within the 10 s trace in our earlier microinfusion studies either (Pezze et al., 2015, 2017). In the present study, the increase in responding within the 2 s trace was less marked in rats treated with scopolamine. Similarly, scopolamine‐treated rats responded overall less in the 10 s ISI but without any evidence consistent with an effect on timing. In the 10 s trace, the effect of bins arose because there was some relatively increased responding toward the middle to later portion of the trace interval, but responding was not increased in the final bin suggesting that timing was not fully developed. Moreover, (the acquisition of) the distribution of responding within the 10 s trace was unaffected by drug (which was significant only as a main effect). Thus, it seems that the use of a procedure optimized for microinfusions in effect precluded examination of (effects on) timing.

### 4.3 The role of additional conditioning mechanisms

In addition to the possibility that effects on timing could have been identified if the trials had been distributed over a greater number of days, there are other underlying cognitive mechanisms which the present study cannot distinguish. We have focused on CS responding as the measure of associative learning in the present task. However, if as we would wish to argue, the ISI is discriminated from the ITI, the procedure in use may amount to sequential conditioning (Baeyens, Vansteenwegen, Hermans, Vervliet, & Eelen, 2001). As shown in Figure 3a, responding in the 2 s ISI progressively increased over the 4 days of conditioning consistent with the development of anticipatory responding in the trace interval as well as to the CS.

Moreover, there is good evidence to suggest that the learning of sequential discriminations results in occasion setting. In other words, rather than the CS eliciting the nose‐poking CR because it signals the occurrence of the US, it may rather set the occasion for responding in the subsequent trace interval (Holland, 1989, 1992). Contexts (such as that provided by the ITI in the present study) are more typically viewed as occasion setters for CS‐US relations (Bouton & Swartzentruber, 1986). Furthermore, just as the same experimental stimulus can have the role of both CS and occasion setter (Holland, 1992), the same has been suggested to apply for contextual cues (Bouton, 2010). In the present study, the differences in responding show that the mini‐context of the ISI trace is functionally different from the ITI; the context predicts the US only on the specific occasions when it has been signaled by the preceding CS. Thus, effects of intra‐hippocampal scopolamine mediated by occasion setting are not excluded.

### 4.4 Selectivity of the microinfusion procedure

With respect to the selectivity of the microinfusion procedure, the expected spread of a 0.5 µl injection is around 1 mm (Allen et al., 2008; Bast & Feldon, 2003; Myers, 1966). The minimally pharmacologically active concentration of scopolamine beyond the immediate spread of the bolus and wider diffusion radius is unknown, and the drug infusion may also have spread up the cannula tract (Wise & Hoffman, 1992). However, dorsal hippocampus spans a relatively large area, extending around 3 mm in the mediolateral plane for example (Paxinos & Watson, 1998). Moreover, the area is covered with fibers, which limit diffusion to extra‐hippocampal structures (Morris, Halliwell, & Bowery, 1989). Diffusion to ventral hippocampus can similarly be excluded based on likely spread of the 0.5 µl injection volume and its relative positioning.

It is therefore reasonable to assume that we have examined the effects of scopolamine in (part of) dorsal hippocampus. This distinction matters given the compelling body of evidence for functional differentiation along the septotemporal axis of the hippocampus (Bannerman et al., 2004; Bast & Feldon, 2003; Strange, Witter, Lein, & Moser, 2014). Moreover, electrophysiological evidence has confirmed relatively greater neuronal activation in dorsal than in ventral hippocampus during CS presentations and the trace interval of an eyeblink conditioning procedure (Weible et al., 2006). On this basis, the effects of scopolamine observed in the present study would not be expected to reproduce following infusion restricted to ventral hippocampus. Though since excitotoxic lesions extending also to include ventral hippocampus similarly impair aversive trace conditioning (Bangasser et al., 2006), there are no grounds to suppose that any spread to ventral hippocampus should counteract the effects of infusion in dorsal hippocampus.

Although not directly examined in the present study, a role for ACh nicotinic receptors should also be considered. In appetitive conditioning procedures similar to those used in the present study, systemic nicotine was without effect on 10 s trace conditioning but a 2 s interval was not examined (Cassaday et al., 2008). In conditioned freezing procedures, systemic nicotine‐enhanced trace conditioning in mice (Gould, Feiro, & Moore, 2004) and the key brain substrates mediating nicotinic ACh receptor modulation of trace conditioning have been found to include hippocampus as well as mPFC (Raybuck & Gould, 2010).

### 4.5 Generality of the findings

With respect to the generality of the findings, we did not directly compare the effects of scopolamine in aversive conditioning in the present study. However, using the identical appetitive behavioral procedure, Pezze et al. (2017) compared the effects of systemic scopolamine in aversive trace conditioning procedure. Since systemic scopolamine was without effect in the aversive procedure, we went on to examine the effects of microinfusions (in this case in mPFC) using the appetitive procedure only. The same rationale was applied in the present study without further use of animals in the aversive variant. However, scopolamine microinfused in hippocampus might nonetheless have had an effect on aversive trace conditioning. Indeed, Pang et al. (2010) reported just such an effect with scopolamine infusions in dorsal hippocampus (30 µg/side).

As a first step, and in common with the other studies conducted within the same project, the present study used male Wistar rats. There have been few reports of sex differences in appetitively motivated procedures (Prendergast, Onishi, & Zucker, 2014). Future studies should also include females to rectify this omission, particularly if further comparisons with aversively motivated procedures are to be drawn. Although in aversively motivated behaviors there is evidence that stage of the estrous cycle results in behavioral variability in rodents, meta‐analysis of 293 studies suggests that estrous cycle makes little or no difference to the performance of exploratory or appetitive tasks; indeed, behavioral variability may even be greater in males (Prendergast et al., 2014).

### 4.6 Implications

The role of ACh in trace conditioning is consistent with other evidence for its role in attention (Sarter, Parikh, & Howe, 2009), learning (Hangya, Ranade, Lorenc, & Kepecs, 2015), working memory (Teles‐Grilo Ruivo et al., 2017), and reward (Hangya et al., 2015). Trace conditioning already shows good translational validity as model of hippocampal‐dependent memory function (Bangasser et al., 2006; Sweatt, 2004). The present study extends on these findings to suggest the further potential of cholinergic treatments, to target muscarinic receptors as a treatment strategy for working memory enhancement, selectively in dorsal hippocampus when regional activation is feasible (although nicotinic receptors may also play some role; Gould et al., 2004; Raybuck & Gould, 2010). Neuromodulatory effects of ACh in cortical regions may also be important (Hasselmo, 1999; Sarter et al., 2009; Teles‐Grilo Ruivo et al., 2017). Indeed, using the same appetitive trace conditioning procedures, we have found a similar profile of neuromodulation after microinfusion in mPFC (Pezze et al., 2017). Flesher, Butt, and Kinney‐Hurd (2011) systematically compared ACh release measured by microdialysis probes in hippocampus and mPFC during appetitive trace and delay conditioning. The results showed that ACh release in both structures was correlated with trace but not delay conditioning performance (Flesher et al., 2011). The similarity between the effects of scopolamine in hippocampus and mPFC is also consistent with evidence for the coordination of tonic ACh release in dorsal hippocampus and mPFC during performance of a rewarded working memory task (Teles‐Grilo Ruivo et al., 2017). In future research, such functional interactions should be further investigated using disconnection procedures, to compare the effects of bilateral infusion of scopolamine in hippocampus and mPFC (Pezze et al., 2017) with those of unilateral infusion of scopolamine in hippocampus combined with the contralateral infusion in mPFC.

## CONFLICT OF INTEREST

The authors have no competing financial interests and nothing to disclose.
